# Chloroform and Morphia in Maniacal Chorea

**Published:** 1908-12-19

**Authors:** 


### CHLOROFORM AND MORPHIA IN MANIACAL CHOREA.

It happens now and then that chorea is so violent that some urgency treatment is required upon altogether different lines to the routine treatment which the case will require when the immediate violence of the attack has declined a little. The nursing and the padded sides to the bed to prevent self-injury are obvious. It is to the medicinal treatment that we would refer; and a suggestion made by Dr. P. Churton, in a letter from Leeds to the *British Medical Journal*, in which he advocates the combined use of morphia and chloroform in these cases, seems well worthy of consideration and trial. Dr. Churton's remarks apply in the main to active mania of all sorts, whether it be maniacal chorea or not. He points out that doses of morphia or of hyoscine, so large as to be bordering on the unsafe, often fail to procure sleep in these patients, or even to calm them. Chloroform naturally checks the violence of the bodily movements after the temporary exacerbation during the first few minutes of its administration; but it is scarcely possible to continue giving chloroform indefinitely, and yet, if chloroform alone is relied on, the violent movements all begin again as soon as the anæsthesia passes off. Very different are the results of giving the two drugs together, the morphine hypodermically and the chloroform by inhalation. The chloroform seems to cause the morphine to act with additional rapidity and effect, so that when the anæsthetic is discontinued the morphia continues the sleep in a way which is of the greatest benefit to the patient, preventing exhaustion and accelerating cure. As Dr. Churton, in speaking of maniacal chorea, says: "From chloroform alone the patient would have awakened in a few minutes, and morphine is slow to initiate sleep; on the other hand, during twenty years in which I have made observations upon the combination of the two drugs there has been no case of failure to obtain sleep. It has always seemed that the more violent the patient was the more quickly and easily the chloroform took effect. In severe chorea a long and unbroken sleep appears to be necessary for rapid cure. If the sleep lasts only two or three hours the dose of morphine is increased and the chloroform repeated. In one very violent case the patient awoke calm and conscious after a sleep of nine hours, brought about by $\frac{1}{2}$ grain of morphine subcutaneously, followed by chloroform."

